# Hepatocyte Nuclear Factor 1A (HNF1A) as a Possible Tumor Suppressor in Pancreatic Cancer

**DOI:** 10.1371/journal.pone.0121082

**Published:** 2015-03-20

**Authors:** Zhaofan Luo, Yanan Li, Huamin Wang, Jason Fleming, Min Li, Yaan Kang, Ran Zhang, Donghui Li

**Affiliations:** 1 Department of Gastrointestinal Medical Oncology, The University of Texas MD Anderson Cancer Center, Houston, Texas, United States of America; 2 Department of Pathology, The University of Texas MD Anderson Cancer Center, Houston, Texas, United States of America; 3 Department of Surgical Oncology, The University of Texas MD Anderson Cancer Center, Houston, Texas, United States of America; 4 The Vivian L. Smith Department of Neurosurgery, The University of Texas Medical School, Houston, Texas, United States of America; University of Florida, UNITED STATES

## Abstract

**Background:**

HNF1A (Hepatocyte nuclear factor 1 alpha) is a transcription factor that is known to regulate pancreatic differentiation and maintain homeostasis of endocrine pancreas. Recently, genome-wide association studies have implicated *HNF1A* as a susceptibility gene for pancreatic cancer. However, the functional significance and molecular mechanism of *HNF1A* in pancreatic carcinogenesis remains unclear.

**Methods:**

Using RT-PCR, Western blot and immunohistochemistry methods, we examined *HNF1A* gene expression in eight pancreatic carcinoma cell lines and in paired tumor and normal tissue samples from patients with resected pancreatic ductal adenocarcinoma. We knocked down the *HNF1A* gene expression in two cancer cell lines using three siRNA sequences. The impacts on cell proliferation, apoptosis, and cell cycle as well as the phosphorylation of Akt signaling transduction proteins were examined using ATP assay, flow cytometry and Western blot.

**Results:**

*HNF1A* was expressed in three out of eight pancreatic adenocarcinoma cell lines and the level of *HNF1A* mRNA and protein expression was significantly lower in tumors than in normal adjacent tissues by both RT-PCR and Western Blot analyses. Immunohistochemistry revealed that the level of *HNF1A* expression was significantly lower in tumor tissues than in non-tumor tissues. Selective blocking of *HNF1A* by specific siRNA conferred a 2-fold higher rate of cell proliferation, 20% increased S phase and G2 phase cells, and 30-40% reduced apoptosis in pancreatic cancer cell lines. We further demonstrated that *HNF1A* knockdown activated Akt and its downstream target, the mammalian target of rapamycin (mTOR) in pancreatic cancer cells.

**Conclusion:**

These observations provide experimental evidence supporting a possible tumor suppressor role of HNF1A in pancreatic cancer.

## Introduction

Recent post GWAS (genome-wide association study) data analyses have shown that *HNF1A* (*Hepatocyte nuclear factor 1*) gene variants are associated with risk of pancreatic cancer [1–3]. However, the functional significance and molecular mechanisms of HNF1A-mediated pancreatic carcinogenesis remains unclear.

HNF1A belongs to the homeobox protein family and is an essential transcription factor for many hepatic genes involved in detoxification, homeostasis and metabolisms of glucose, lipid, steroid and amino acid [4]. In addition, HNF1A is an important component of the transcriptional networks governing embryonic pancreas development and differentiation [5], [6]. as well as maintaining the growth and function of islet β cells in adult [7]. Germline heterozygous mutations of *HNF1A* have been found responsible for type 3 MODY (maturity-onset diabetes of the young)[8]. Mutations or common variants of *HNF1A* gene have also been associated with risk of type II diabetes [9–11].

As a transcriptional factor, HNF1A has also been shown to affect intestinal epithelial cell growth and cell lineages differentiation [12], [13] and regulate the expression of microRNA-194 [14]. Previous studies in other human cancers have suggested a tumor suppressor role of *HNF1A* gene. For example, biallelic somatic alterations of *HNF1A* were present in 60% of hepatocellular adenomas and in rare cases of hepatocellular carcinomas in non-cirrhotic liver [15]. *HNF1A* silencing by siRNA in hepatocellular carcinoma cells induced overexpression of several genes encoding growth factor receptors, components of the translational machinery, cell cycle, and angiogenesis regulators [16]. Mutations of *HNF1A* gene were also detected in colorectal cancer with microsatellite instability [17] and in endometrial cancer [18]. Polymorphic variants of *HNF1A* gene have been associated with circulating level of C reactive protein (CRP), a biomarker of inflammation [19], [20]. A recent GWAS study of human N-glycome identifies HNF1A as a master regulator of plasma protein fucosylation [21]. This evidence suggests that HNF1A could play a role in cancer development through regulation of immunity, inflammatory response, and protein folding, as well as cell growth and differentiation. However, there is yet no information on the expression or mutation status and the potential role of *HNF1A* in human pancreatic cancer.

In this study, we aim to demonstrate the expression of *HNF1A* gene in human pancreatic cancer and the impact of *HNF1A* deregulation on cell proliferation, cell cycle, apoptosis and signaling transduction in pancreatic cancer cells.

## Materials and Methods

### Cell lines and Human Tissues

Human pancreatic adenocarcinoma cell lines AsPC-1, Panc-1, MiaPaCa-2, Hs766T, and BxPC-3 cells were purchased from the American Type Culture Collection and cultured as described in their product information sheets. Panc-28, Colo357 and its fast growing (FG) subline, as well as the immortalized normal human pancreatic ductal epithelial (HPDE) cell line were gifts from Drs. Craig D. Logsdon (MD Anderson Cancer Center, Houston, TX)[22], [23]. All cell lines have been authenticated by testing 14 polymorphic markers. Cancer cells were cultured in RPMI 1640 medium or Dulbecco’s modified Eagle’s medium supplemented with 10% fetal bovine serum. HPDE cell was maintained in keratinocyte serum-free medium supplemented with epidermal growth factor and bovine pituitary extract.

Formalin fixed paraffin-embedded (FFPE) sections and frozen samples from 48 pairs of surgically resected pancreatic tumor tissues and their adjacent non-tumor tissues were obtained from MD Anderson Tissue Bank. FFPE was used for immunohistochemistry. Frozen tissues were used for RNA and protein extraction. All tissue samples used in this study were residual surgical samples from patients undergoing tumor resection without pre-operative treatment at MD Anderson Cancer Center with a written informed consent signed by each patient. All tissue samples were evaluated by a pathologist (Dr. Wang) to ensure the cellularity of the tumor tissues and the purity of normal adjacent tissues. The study and the consent form were approved by MD Anderson Institutional Review Board.

### Immunohistochemistry (IHC) and Western Blotting

After deparaffinization, tissue sections were subjected to antigen retrieval and endogenous peroxidase activity blocking. The primary antibody used was rabbit HNF1A antibody from EPITOMICS (Burlingame, CA) at a dilution of 1:200. After treatment with the biotinylated secondary antibody, the antibody complex was detected using an avidin-biotin-peroxidase complex solution and visualized using 3,3’-diaminobenzidine (Zymed Laboratories, Inc., San Francisco, CA). A negative control was included in each experiment by omitting the primary antibody. Images were evaluated by an experienced pathologist for staining patterns in different types of pancreatic cells, different cellular components, as well as in tumor and normal tissues. Sections from the same tissue block was also stained for phosphorylated AKT and mTOR using anti-phospho-AKT (Ser473) and anti-phospho-mTOR (Ser2448) antibodies. The staining intensity was scored as 0 for negative, 1 for weak, 2 for intermediate, and 3 for strong staining. The percentage of cells with positive staining were scored as 0 for none, 1 for 1–50%, and 2 for >50%. The final staining score was the product of the intensity and percentage scores. The difference in staining scores of the non-tumor and tumorous tissues was compared by paired t test.

Protein expression was also evaluated in protein samples extracted from cell lines and frozen tissue samples using Western blotting. Protein was extracted from whole-cell protein lysates and concentration determined using the Bradford staining method. Protein extracts were fractionated by polyacrylamide gel electrophoresis and transferred to a Mini PVDF membrane using a Trans-Blot Turbo transfer system (Bio-Rad Laboratories, Hercules, CA). The primary antibodies used include: HNF1A from BD Biosciences (San Jose, CA) at 1:1000 dilution; total AKT, phospho-AKT (Ser473), and phospho-AKT (Thr308) at 1:2000 dilution; and total mTOR and phospho-mTOR (Ser2448) at 1:1000 dilution from Cell Signaling (Danvers, MA). Beta-actin at 1:3000 dilutions was used as the loading control. After incubation with appropriate secondary antibodies conjugated to horseradish peroxidase, the membranes were exposed to ECL Western Blotting detection reagent. Membranes were stripped for 30 minutes at 55°C in a buffer containing 2% SDS, 62.5 mM Tris (pH6.7), and 100 mM 2-mercaptoethanol for staining of multiple proteins. Staining intensities were quantified by densitometric analysis.

### mRNA expression

Total RNA was extracted from cultured cells as well as from 27 pairs of pancreatic tumor and their adjacent non-tumor tissues using Trizol reagent according to manufacturer’s protocol (Life Technologies, Grand Island, NY). Complementary DNA was synthesized from 1.0 μg of total RNA using the cDNA Synthesis Kit (Bio-Rad Laboratories, Hercules, CA). Quantitative RT-PCR (qRT-PCR) was performed in triplicate samples using predesigned primers and probe sets (Hs00167041-m1, Life Technologies, Grand Island, NY). The RT-PCR results were first normalized to the threshold cycle (Ct) of GAPDH, referred to as ΔCt. The fold change in expression of genes in the tumor group compared to that in the normal group was expressed as 2^-ΔΔCt^, in which-ΔΔCt equals the ΔCt of the tumor group minus the ΔCt of the normal group, which was normalized to 1.

### siRNA blocking

Small interfering RNA (siRNA) transfections were performed using transfection reagent according to the manufacturer’s protocol (Life Technologies, Grand Island, NY). Three different siRNA duplexes targeting *HNF1A* gene (NM_000545) were tested. The sequences of the siRNAs are as follows: 5'-CAGUGAGACUGCAGAAGUAtt-3' (*HNF1A* siRNA1), 5'-GGUCUUCACCUCAGACACUtt-3' (*HNF1A* siRNA2), 5'-CACCUGUCCCAACACCUCAtt-3' (*HNF1A* siRNA3). A negative control siRNA or transfection reagent only was used in mock transfections. AsPC-1 and BXPC-3 cells were transfected and cells were harvested 24 h to 96 h after transfection.

### Cell Growth and Proliferation

Cell proliferation was quantified using a ATP assay (Promega, Madison, WI). Briefly, 10,000 cells per well in 96-well plates were transiently transfected with *HNF1A* siRNA or control siRNA (10 nM). An equal volume of reagent was added to the culture 12, 24, 48 and 72 h after the transfection for luminescence detection. Three independent experiments were conducted at each time point.

### Cell Cycle and Apoptosis assay

72h after transfection, cells were harvested and suspended in citrate stabilizing buffer containing 125 μg/mL propidium iodide (PI) and RNase A. Cell cycle distribution was determined by flow cytometry.

Apoptosis was assessed using the fluorescein isothiocyanate (FITC) annexin V and propidium iodide (PI) dual staining method 72h after transfection. Apoptotic cells were identified by flow cytometric analysis using the BD cell analyzer and FCS Express Software—Clinical Edition (BD Bioscience, San Jose, CA). Both early and late apoptotic cells were counted for relative apoptotic changes. All experiments were performed in triplicates.

All data in replicate experiments were described as mean ± SD. Paired t test or student t test (2-tailed) were applied with *P* value < 0.05 as the significance level.

## Results

### Expression of *HNF1A* in pancreatic carcinoma cell lines

**Fig 1:**
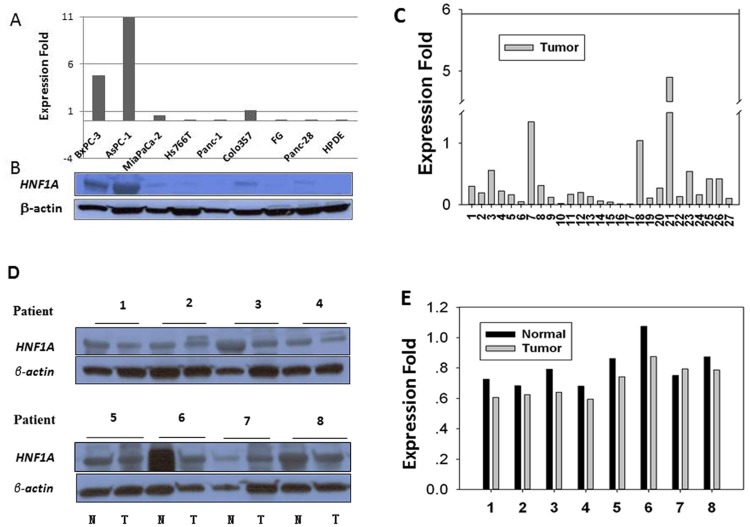
HNF1A expression in human pancreatic carcinoma cell lines (A and B), and human pancreatic tissues (C and D). A, mRNA expression level in human pancreatic cancer cell lines relative to control cell line COLO357 as measured by qRT-PCR. B, protein expression in corresponding cell lines by Western blot. C, fold differences in mRNA expression levels of 27 pancreatic tumors compared to their paired adjacent non-tumor tissues. D and E, Western blots and quantitative measurement of expression of HNF1A protein in eight paired human pancreatic tumors samples (T) and their adjacent non-tumor (N) tissues. The expression levels of HNF1A are normalized to those of β-actin.

Using quantitative RT-PCR (qRT-PCR) and Western blot analyses, we showed that *HNF1A* was expressed in three well-characterized pancreatic carcinoma cell lines derived either from the primary pancreatic adenocarcinoma tumors (BxPC-3) or metastatic sites (AsPC-1, Colo357) with the highest level detected in AsPC-1 (Fig. 1A). *HNF1A* expression was barely detectable in MiaPaCa-2 cells and not detectable in the remaining carcinoma cell lines and HPDE (Fig. 1). Western blot analysis confirmed the presence of HNF1A protein in BxPC-3, AsPC-1 and Colo357 cells, the lower level in MiaPaCa-2 and the absence in Panc-1, FG, Hs766T, Panc-28 and HPDE cells (Fig. 1B).

### Reduced expression of *HNF1A* in human pancreatic adenocarcinoma

*HNF1A* mRNA expression were detected by qRT-PCR in 27 paired resected pancreatic adenocarcinoma tumor tissues and their adjacent non-tumor tissues. The level of *HNF1A* expression was significantly lower in tumor than those in non-tumor tissues (*p* = 0.005, paired *t* test) (Fig. 1C). The mean level of *HNF1A* mRNA expression in tumor tissues was reduced to 44.4% of that in non-tumor tissues, although three out of the 27 pairs showed a higher level of *HNF1A* expression in the tumors.

**Fig 2:**
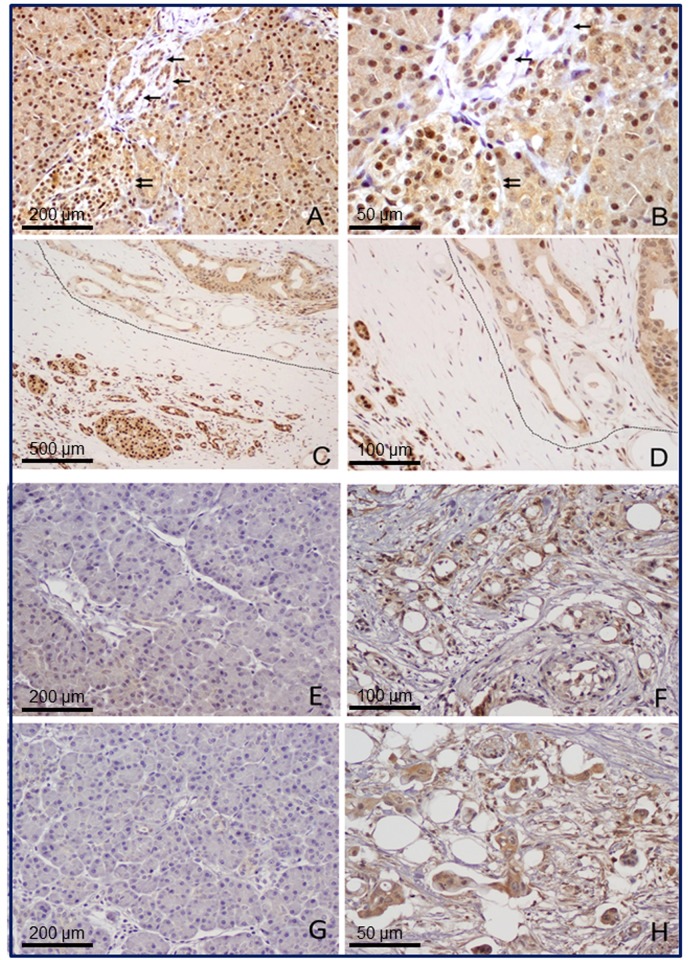
Representative micrographs of HNF1A, pAKT, pmTOR in PDAC cancerous tissue or its surrounding non-tumor tissue by immunohistochemistry. Images (A and B) show cytoplasmic and nuclear expression of HNF1A in normal pancreatic tissue. The normal pancreatic ducts are marked with single arrow. Islet cells are marked with double arrows. Pancreatic islet cells have higher level of HNF1A expression than acinar and ductal cells. Images (C and D) show the expression of HNF1A in pancreatic ductal adenocarcinoma (circled) and benign pancreatic ductal cells and islet cells adjacent to the tumor (left). Panel E-H show the representative micrographs for p-AKT (E and F) and p-mTOR (G and H) expression in normal pancreatic tissues (E and G) and pancreatic ductal adenocarcinoma (F and H). Magnifications: 100X for A, E and G; 400X for B and H, 40X for C, 200X for D and F.

Next, Western blot were conducted in eight pairs of tumor and non-tumor tissues to examine HNF1A expression at the protein level. By Western blot (Fig. 1D), we found a lower level of HNF1A protein in 7/8 (87.5%) of the tumors compared to their adjacent non-tumor tissues (Fig. 1E). The mean (± SD) intensity of the HNF1A band after normalized to that of the β-actin was significantly lower in tumor (0.71 ± 0.11) than those in non-tumor tissues (0.80 ± 0.13) (*p* = 0.005, paired *t* test). The IHC experiments revealed clear differences in HNF1A protein expression between normal (Fig. 2A and 2B) and cancerous pancreas (Fig. 2C and 2D). HNF1A protein was present with a strong intensity in normal endocrine and exocrine pancreas. The islet cell and acinar cell showed a stronger nuclei staining than the ductal cells did. In contrast, in tumorous tissue, HNF1A was expressed at moderate levels in islet and acinar cells but at a low or undetectable level in ductal cells. Stromal tissues surrounding the neoplastic lesions did not show significant staining for HNF1A. The average staining score (mean ± SD) was 5.08 ± 2.17 versus 2.27 ± 1.45 in 48 pairs of non-tumor and tumorous tissues, respectively (*P<*0.001, paired *t* test) (Table 1).

**Table 1:** Expression of HNF1A, pAKT, pmTOR in paired tumor and normal pancreatic tissues.

Marker N	Tumor	Normal	P value
HNF1A 26	2.27 ± 1.45	5.08 ± 2.17	<0.001
pAKT* 28	4.98 ±1.63	3.82 ±0.98	<0.001
pmTOR* 24	5.71 ± 2.18	4.17 ± 1.63	0.001

*IHC used anti-phospho-AKT Ser473 (pAKT) and anti-phospho-mTOR Ser2448 antibody (pmTOR).

### Blocking *HNF1A* by specific siRNA

**Fig 3:**
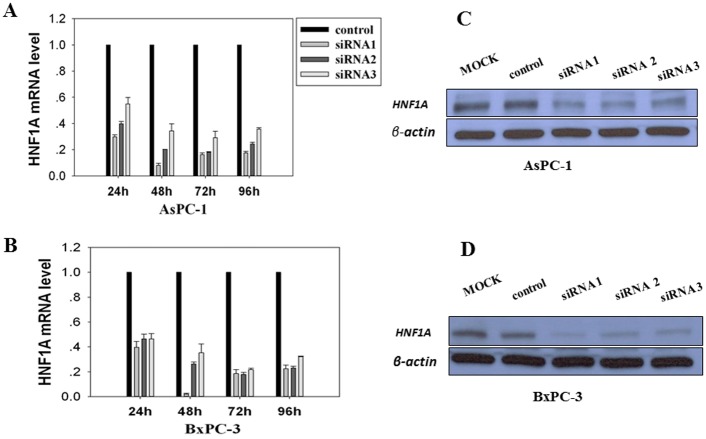
HNF1A knockdown by specific siRNAs in AsPC-1 (A) and BxPC-3 (B) cells. Cells were transfected independently with three different sequences of siRNA directed against HNF1A (siRNA1, siRNA2, siRNA3), or with a control siRNA (Control). Inhibition efficiencies were assessed by qPCR for relative levels of HNF1A mRNA at 24 h to 96 h following the transfections. Western Blot analyses of AsPC-1 and BxPC-3 cells transfected with anti-HNF1A siRNAs. Data are shown for 96 h following transfections. Expression of β-actin was used as an internal control.

Three different siRNAs sequences targeting *HNF1A* exon 4 (siRNA1), exon 1 (siRNA2) or exon 8–9 junction (siRNA3) were transfected into AsPC-1 and BxPC-3 cells. Quantitative PCR analysis showed that 48h after treatment, the three independent siRNA-mediated *HNF1A* knockdowns resulted in 92%, 80% and 64% extinction of *HNF1A* mRNA expression in AsPC-1 cells, and 98%, 74% and 65% extinction in BxPC-3 cells, respectively (Fig. 3A and 3B). Complementing the mRNA data, Western blot analysis showed that expression of HNF1A protein in cells treated with specific siRNAs was substantially reduced (Fig. 3C and 3D). On the other hand, transcription level of *HNF1A* was not affected in vector controls. Based on these observations, we used siRNA1 and siRNA2 in the following experiments.

### Impact of *HNF1A* knockdown on cell growth and apoptosis

**Fig 4:**
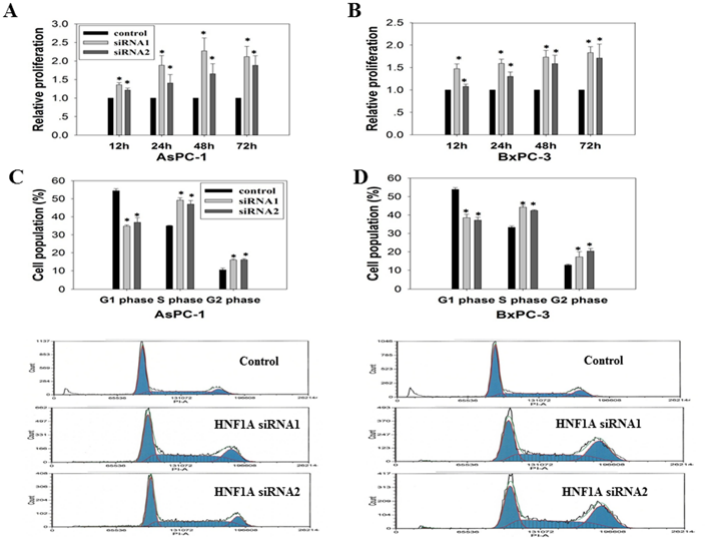
Effect of HNF1A knockdown on cell proliferation and cell cycle. Pancreatic cancer cell lines were transfected with one of the two HNF1A siRNAs or a control siRNA. The proliferation rate of the siRNA-transfected cells was assessed by CellTiter-Glo assay on AsPC-1 (A) and BxPC-3 (B) cells 12 h to 72h after transfection. Cell cycle distribution was determined by Fluorescence-activated cell sorting analysis in AsPC-1(C) and BxPC-3 (D) cells. Cells were stained with PI, and analyzed for DNA content using flow cytometry 72h following transfections. The populations of cells in G1, S, and G2 phases are characterized by different intensities of PI-A fluorescence and are indicated. Data are shown relative to the control. *P<0.05 compared with each corresponding control.

**Fig 5:**
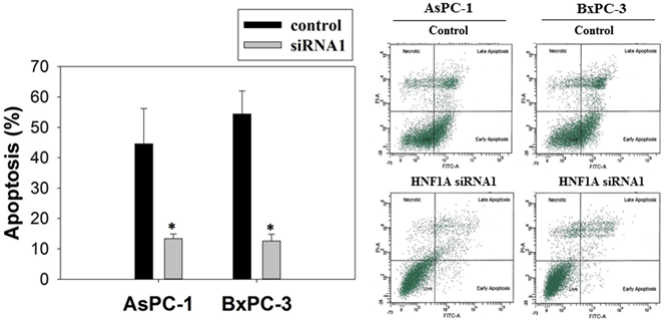
Effect of *HNF1A* knockdown on apoptosis. 72 h after transfection with anti-*HNF1A* siRNAs, the AsPC-1 and BxPC-3 cells were stained with FITC conjugated Annexin-V/PI. Percentage of Annexin-V/PI-stained cells was determined by flow cytometry. Dot plot was divided into a quadrant: (1) necrotic cells, (2) late apoptotic cells, (3) living cells, and (4) early apoptotic cells. Bar graph shows the percentage of apoptotic cells. Values are expressed as mean ± SD from three different experiments. *P<0.05 compared with the control.

Using siRNA1 and siRNA2 as an efficient tool for silencing the *HNF1A* gene, we found that *HNF1A* knockdown conferred a 2-fold increased proliferation of AsPC-1 (Fig. 4A) and BxPC-3 (Fig. 4B) cells. Notably, cell growth was not affected by the transfection of vector control. Consistently, we observed that *HNF1A* knockdown lead to a 20% reduced G0/G1 population and a 20% increased S/G2 phase population in the two tested cell lines (Fig. 4C and 4D). In addition, comparing to the vector control, transfection with *HNF1A* siRNAs significantly decreased the percentage of apoptotic cells. The Annexin-positive cells (mean ± SD) was reduced from 44.57 ± 11.57 to 13.37 ±1.51 in Aspc-1 cells, and from 54.37 ± 7.58 to 12.6 ± 2.19 in BxPC-3 cells (Fig. 5).

### Loss of *HNF1A* actives the AKT/mTOR signaling pathway

**Fig 6:**
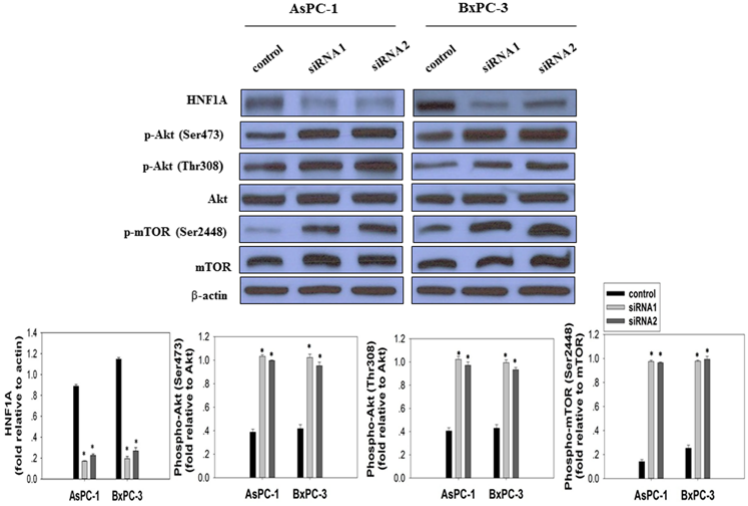
Effect of *HNF1A* knockdown on AKT/mTOR signaling pathway was assessed by Western Blot. AsPC-1 and BxPC-3 cells were transfected with control siRNA and two different *HNF1A* siRNAs as indicated. 96h after transfection, cell lysates were immunoblotted with anti- HNF1A antibody, anti-phospho-AKT Ser473 antibody, anti-phospho-AKT Thr308 antibody, and anti-phospho-mTOR Ser2448 antibody. Membranes were stripped and re-probed with anti-AKT antibody, anti-mTOR antibody and anti-β-actin antibody. The fold differences in protein expression levels of cells transfected with *HNF1A* siRNA and control siRNA was presented as mean ± SD from three independent experiments.

The AKT/mTOR signaling pathway is crucial to many aspects of cell growth, survival, and apoptosis [24]. The constitutive activation of AKT/mTOR has been involved in the pathogenesis and progression of pancreatic cancer [25]. To elucidate the molecular mechanisms modulated by *HNF1A* inactivation in pancreatic cancer cells, we investigated the impact of *HNF1A* knockdown on AKT/mTOR signaling pathway. Using Western blotting, we found that oncogenic signaling phosphor-AKT and its downstream phosphor- mTOR were up-regulated after transfection with *HNF1A* siRNAs in both AsPC-1 and BxPC-3 cells (Fig. 6). Accordingly, the levels of phosphorylation of AKT at Ser473 and Thr308 were significantly increased compared with their corresponding control. Concomitantly, the degrees of mTOR Ser2448 phosphorylation were remarkably enhanced. No effect was observed on total AKT and mTOR expression. IHC analysis in paired tumor and normal pancreatic tissues also found significantly higher levels of phosphorylated AKT and mTOR in tumors than in normal tissues (Fig. 2, Table 1).

## Discussion

In this study, we have provided some experimental evidence that *HNF1A* gene may act as a tumor suppressor in pancreatic cancer. First, reduced expression of *HNF1A* was detected in human pancreatic adenocarcinoma at both mRNA and protein levels. Second, *HNF1A* knockdown in pancreatic cancer cells lead to increased cell proliferation and reduced apoptosis. Third, inhibition of *HNF1A* enhanced the activation of AKT-mTOR signaling pathway. These data are consistent with previously reported observations in liver cancer studies [15], [16]. supporting a tumor suppressor role of *HNF1A* in the development of human cancers.

*HNF1A* gene expression was initially discovered in the liver, subsequently shown to be expressed in the epithelia of several organs including pancreas, kidney and intestine [13]. *HNF1A*-deficient mice (*HNF1A* ^*-/-*^) are born normally, but suffer from hepatic, pancreatic and renal functional defects [26–28]. In humans, patients carrying mono-allelic mutations in *HNF1A* suffer from type 3 MODY with renal dysfunctions [8]. Somatic mutations of *HNF1A* gene have been reported in hepatoma, colon cancer and endometrial cancer [16–18]. Recent GWAS studies have suggested that *HNF1A* gene is associated with risk of pancreatic cancer [1–3], which prompted the current investigation on the functional significance of this gene in pancreatic cancer.

In this study, IHC analysis of human pancreatic tissues have shown that HNF1A protein was expressed at a higher level in islet cells and acinar cells compared to that in ductal epithelial cells, which is consistent with the functional significance of this gene in maintaining the homeostasis of endocrine pancreas. On the other hand, the mRNA and protein expression level of HNF1A (by both IHC and Western blot analyses) was significantly lower in pancreatic tumors than in the surrounding non-tumor tissues, which is consistent with a potential tumor suppressor role of this gene in pancreatic cancer.

The tumor suppressor role of HNF1A is also supported by findings from the gene knockdown experiments. We have observed that inhibition of *HNF1A* by siRNAs in pancreatic carcinoma cells resulted in a significantly increased cell proliferation. Similar results have previously been reported by Molero X et al that HNF1A^-/-^ mice display increased acinar cell proliferation in basal conditions and after pancreatitis induction [29]. To better understand the mechanism of *HNF1A*-inactivation induced cell growth, we have further shown that down-regulation of *HNF1A* resulted in a decreased G0/G1 phase, an increased S phase, and a mild increase of G2/M phase fraction. A previous study conducted in hepatoma cell lines has found elevated level of cyclin D1 in response to *HNF1A* inactivation [16]. Further study is needed to demonstrate which cell cycle regulator modulates the *HNF1A* siRNA-mediated proliferation in pancreatic cancer cell lines.

We next showed a reduced apoptosis rate in *HNF1A*-inactivated pancreatic carcinoma cells. This effect seems in contradiction with observations made in human pancreatic beta cells. Several previous studies have shown that over-expression of a dominant-negative mutant of *HNF1A* (DN-HNF1A) resulted in decreased phosphoinositide-3 kinase (PI-3K)/Akt activity, thereby attenuating cell growth and proliferation and sensitizing beta cells to apoptosis [28],[30–32]. This discrepancy might result from difference in cell and pathological process, and show the complex effect of *HNF1A* in different types of cells and pathological processes.

We further elucidated the downstream signaling pathways that HNF1A exerts its tumor suppressor function in pancreatic cancer and found that *HNF1A* knockdown activated Akt/mTOR signaling pathway. The PI3K/Akt/mTOR signaling axis plays a critical role in regulating cell proliferation, apoptosis, angiogenesis and metastasis, which is central to the development and maintenance of cancer cells. Aberrant PI3K/Akt signaling is common in pancreatic cancer [33], [34]. Our data showed that *HNF1A*-inactivation significantly increased the levels of Akt Ser473 and Thr308 phosphorylation and mTOR 2248 phosphorylation. It is known that phosphorylation of Akt at Thr 308 can regulate protein synthesis and cell proliferation whereas phosphorylation of Akt at Ser 473 is associated with resistance to apoptosis by controlling subcellular localization of pro-apoptotic proteins [35]. It is known that mTOR, a serine-threonine kinase, is regulated by phosphorylation on Ser2448 in response to PI3K/Akt oncogenic signaling. mTOR regulates cell growth, cell proliferation, cell motility, cell survival, protein synthesis, and transcription. Therefore it is possible that the impact of *HNF1A*-inactivation on cell growth and apoptosis are, at least in part, via the mechanism of activation of AKT/mTOR signaling pathway.

Chronic pancreatitis is a known risk factor for pancreatic cancer. Genes regulating pancreatic regeneration may also be involved in the development of chronic pancreatitis, and pancreatic carcinogenesis. A recent study conducted in a pancreatitis animal model has shown an injury-responsive down-regulated expression of *HNF1A* in acinar cells [29], which was related to an increased acinar cell proliferation and reduced digestive enzymes. Apparently HNF1A controls tissue-specific transcriptional programs because it enhances cell growth in pancreatic islets but suppresses cell proliferation in hepatocytes [36]. The inhibition of cell proliferation in acinar cells and ductal epithelial cells could be one of the major mechanisms that linking HNF1A in pancreatic carcinogenesis. Another recent study has also shown that overexpression of *HNF1A* gene in pancreatic cancer cell lines inhibited cell growth, induced G_0_/G_1_ arrest and apoptosis [37]. These effects correlated with HNF1A-induced down-regulation of cell cycle genes and decreased expression of anti-apoptotic genes [37].

In conclusion, we have shown that *HNF1A* expression is significantly decreased in human pancreatic adenocarcinoma tumors. Selective blocking of *HNF1A* by specific siRNA significantly promoted pancreatic cancer cell proliferation and inhibited apoptosis in vitro. We have further revealed that *HNF1A* knockdown activates Akt/mTOR signaling pathway in pancreatic cancer cell lines. These findings support a potential tumor suppressor role of *HNF1A* in pancreatic cancer.
